# Giant Multinucleated Cells in Aging and Senescence—An Abridgement

**DOI:** 10.3390/biology11081121

**Published:** 2022-07-27

**Authors:** Malgorzata Kloc, Ahmed Uosef, Arijita Subuddhi, Jacek Z. Kubiak, Rafal P. Piprek, Rafik M. Ghobrial

**Affiliations:** 1 The Houston Methodist Research Institute, Transplant Immunology, Houston, TX 77030, USA; auosef@houstonmethodist.org (A.U.); asubuddhi@houstonmethodist.org (A.S.); rmghobrial@houstonmethodist.org (R.M.G.); 2 The Houston Methodist Hospital, Department of Surgery, Houston, TX 77030, USA; 3 Department of Genetics, MD Anderson Cancer Center, The University of Texas, Houston, TX 77030, USA; 4 Dynamics and Mechanics of Epithelia Group, Faculty of Medicine, Institute of Genetics and Development of Rennes, University of Rennes, CNRS, UMR 6290, 35043 Rennes, France; jacek.kubiak@univ-rennes1.fr; 5 Laboratory of Molecular Oncology and Innovative Therapies, Department of Oncology, Military Institute of Medicine, 04-141 Warsaw, Poland; 6 Department of Comparative Anatomy, Institute of Zoology and Biomedical Research, Jagiellonian University, 30-387 Krakow, Poland; rafal.piprek@uj.edu.pl

**Keywords:** aging, senescence, senescence-associated secretory phenotype, SASP, cell fusion, multinucleation, giant cells, macrophages, arteries

## Abstract

**Simple Summary:**

Aging is a progressive decline of an organism over time. In contrast, senescence occurs throughout an organism’s lifespan. It is a cell-cycle arrest preventing the proliferation of damaged cells. Cellular and molecular senescence timing is crucial for the pace of aging and disease development and progression. The accumulation of senescent cells during a lifespan leads to organismal senescence. Senescent multinucleated giant cells are present in many age-related diseases and cancer. Although senescence was assumed to be irreversible, studies now show that senescent multinucleated giant cells overcome senescence in various cancers, becoming the source of highly aggressive mononucleated stem-like cells, which divide and initiate tumor development and progression.

**Abstract:**

This review introduces the subject of senescence, aging, and the formation of senescent multinucleated giant cells. We define senescence and aging and describe how molecular and cellular senescence leads to organismal senescence. We review the latest information on senescent cells’ cellular and molecular phenotypes. We describe molecular and cellular features of aging and senescence and the role of multinucleated giant cells in aging-related conditions and cancer. We explain how multinucleated giant cells form and their role in aging arteries and gonads. We also describe how multinucleated giant cells and the reversibility of senescence initiate cancer and lead to cancer progression and metastasis. We also describe molecules and pathways regulating aging and senescence in model systems and their applicability to clinical therapies in age-related diseases.

## 1. Introduction

### Aging Versus Senescence

Although the terms “aging” and “senescence” are often used interchangeably, this is incorrect because they describe entirely different processes. Aging is a progressive decline by accumulating detrimental changes in an organism over time. In contrast, senescence occurs throughout an organism’s lifespan. It refers to cell-cycle arrest, preventing the proliferation of damaged cells and failure to resume the cell cycle in response to mitogenic stimuli. There is also a difference between cellular and organismal senescence. Senescent cells accumulate during the lifespan, leading to the aging of whole organisms, i.e., organismal senescence. Senescence is also different from quiescence, a reversible cell-cycle arrest [1]. Cellular and molecular senescence timing determines the pace of aging and disease development. Senescence was first described by Hayflick and Moorhead [2] in cells at the end of their replicative lifespan affected by telomere shortening [3]. In addition to telomere shortening and disorder, various stimuli, such as DNA damage, oxidative stress, oncogenes, and viral and bacterial infection, induce senescence [4,5,6,7,8]. It is a common belief that senescence evolved as a tumor-suppressive mechanism preventing the malignant transformation of damaged cells [9,10]. However, developmentally programmed senescence, regulating embryonic development and patterning (for example, limbs, kidneys, and neural system), suggests that non-pathological senescence must be evolutionarily older.

Recent studies show that approximately 60% of in vitro fertilized human embryos are arrested at the 3–8 cell stage due to a senescence-like state. These embryos have lower ribosomes, histones, MYC, and p53 activity. Some of them also have decreased glycolysis or higher- or lower-than-normal oxidative phosphorylation. Embryos treated with the SIRT agonists, resveratrol or nicotinamide riboside (NR) change their metabolism, partially reversing senescence [11].

Senescence is one of the causative agents of aging. Senescent cell numbers increase with age, thus contributing to organismal senescence and aging-related disorders. However, senescence is also beneficial as it is crucial for embryogenesis, tissue homeostasis, remodeling, and wound healing [4,12]. Although, by definition, senescence permanently arrests the cell cycle, recent studies indicate that this is not necessarily true and that, in specific biological/pathological contexts, senescence may be reversible [13,14,15]. Experimental data show that eliminating senescent cells by pharmacologic or genetic methods improves health and prolongs life. The main factors driving senescence during aging are: epigenetic, telomere attrition, DNA and mitochondrial damage, changes in protein synthesis and response to nutrient signaling, exhaustion of stem cells, and chronic inflammation [16].

Senescent cells have changed morphology, organelle functions, chromatin organization, gene expression, and metabolism, resulting in the acquisition of a pro-inflammatory phenotype, known as the senescence-associated secretory phenotype (SASP), characterized by the overexpression of interleukins IL-1a, IL-6, IL-15, and IL-8, and GRO-a, MIP-1a, IFN γ, VEGF, ICAM-1, and GM-CSE chemokines, signaling molecules, and growth factors [15,16,17,18,19,20].

Senescent cells are often flattened and highly enlarged. Such an increase in cytoplasm volume and the cytoplasm to DNA ratio results in the dilution of transcription-required factors leading to the cell-cycle arrest and senescence [21,22]. Senescent cells often form giant multinucleated cells (GMCs), containing many nuclei and vacuoles. Sometimes these giant cells have many large lysosomes and increased senescence-associated beta-galactosidase (SA-βgal) activity [22,23]. The integrity of the nuclear membrane is compromised in giant cells, and nuclear and chromatin architecture is changed because of loss or under-expression of Lamin B1. Lamin is an essential structural component of the nuclear lamina, a network of filaments underlying the nuclear envelope [24,25]. The gene transcription depends on the architecture and condensation status of chromatin. In senescent cells, to silence the transcription of the proliferation-promoting genes, the chromatin condenses into dense senescence-associated heterochromatin foci (SAHFs) [22,26]. The loss of nuclear membrane integrity causes the release of chromatin fragments into the cytoplasm. This activates the DNA-sensing receptor cyclic GMP–AMP synthase (cGAS) and its downstream effector stimulator of the interferon genes (STING) pathway, fueling the development of the SASP phenotype [22,27,28].

Other hallmarks of the senescence phenotype are the changes in the number, size, cristae structure, metabolic properties of mitochondria, and mitostasis [29]. Because of the reduction in mitophagy, the senescent cells have more mitochondria with deteriorated mitochondrial oxidative phosphorylation (OXPHOS). The dysfunction of mitochondria increases the production of reactive oxygen species (ROS), which enhance DNA damage and DNA-damage response pathway (DDR), ultimately aggravating SASP [29,30,31]. Mitochondria of senescent cells are also resistant to apoptosis through the upregulation of the antiapoptotic Bcl-2 proteins and pro-survival pathways [32,33].

Cell-cycle arrest during senescence depends on the decreased phosphorylation of Retinoblastoma protein (pRB). The hypo-phosphorylated RB binds the transcription factor E2F, preventing its binding to the promoters of genes regulating the G1/S phase transition and entry into the S phase of mitosis, ultimately halting the cell cycle. The pRB function, and thus cell-cycle arrest, is regulated by the p16Ink4a/RB and p53/p21CIP1 pathways, whose final downstream effector is the pRB [15,22].

## 2. Giant Multinucleated Cell Formation

Giant cells form through the fusion of individual mononuclear cells. The fusing cells can be of the same or of different origin (homotypic or heterotypic fusion, respectively). Because macrophages are intrinsically fusogenic, they are the primary source of the giant multinuclear cells observed in aging. They derive from either the homotypic or heterotypic fusion of macrophages with other cell types. Cell fusion is a multi-step endeavor. First, cells must be fusion-competent; approach each other; contact, adhere, and merge the cell membranes to become a single, functional entity. The molecules and pathways involved in each step of cell fusion are described in detail by Helming and Gordon [34] and Kloc et al., 2022 [35]. 

## 3. Giant Cells in Aging Arteries

**Figure 1:**
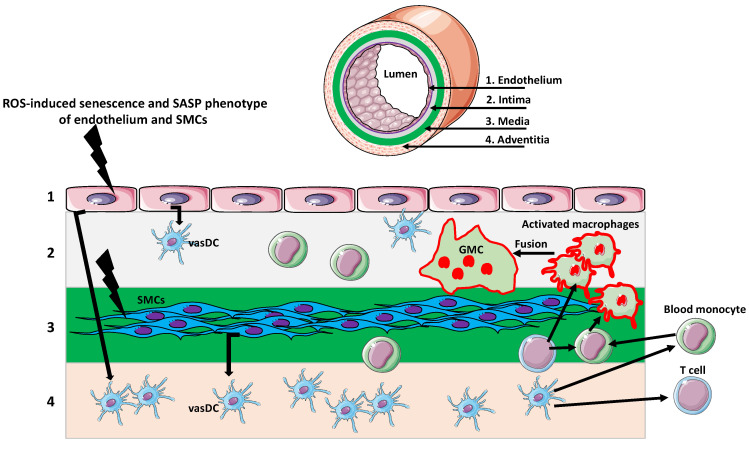
Giant multinucleated cells in giant cell arteritis (GCA).

Arteriosclerosis and giant cell arteritis (GCA) are typical examples of vascular aging. Vascular aging depends on immune cells and inflammaging, a low-grade inflammation resulting from long-term stimulation of the innate immune system [36]. GCA is an inflammatory vasculopathy of large- and medium-sized arteries [36,37,38]. Mitochondrial dysfunction, causing the excessive production of ROS, is a significant contributor to vascular aging. ROS induces senescence of vessel endothelial and smooth muscle cells, which acquire the SASP phenotype [37,39,40]. The first immune cells responding to SASP and its pro-inflammatory secretome are vascular dendritic cells (vasDCs), located in the vessel wall. The cytokines released from the activated DCs recruit T cells and monocytes. Monocytes differentiate into macrophages, which phagocyte cell debris and release various signaling molecules, including VEGF, further enhancing vessel inflammation and over-proliferation of the myofibroblasts in the vessel wall intima [37,41]. Subsequently, the activated macrophages fuse into giant multinucleated cells (GMCs), which produce a massive amount of matrix metalloprotease-9 (MMP-9) and other proteolytic enzymes. The resulting excessive digestion of the extracellular matrix disrupts the vessel wall, allowing infiltration by the T cells, and furthering the inflammation and the formation of the inflammation foci (granulomas). The ultimate result is a collapse of the inner elastic membrane and occlusion of the vessel lumen by overgrown muscle cells (Figure 1) [37,38,42,43].

The artery wall consists of several layers. The endothelial layer (1) faces the vessel lumen and abuts the intima (2), which contains vascular dendritic cells (vasDCs). Below is the media (3) containing smooth muscle cells (SMCs). The outermost layer is adventitia (4) composed of collagenous and elastic fibers matrix and various immune cells, including vasDCs. Different signals originating from age-related cellular, organellar, and metabolic dysfunctions, such as excessive reactive oxygen species (ROS) production, induce senescence and senescence-associated secretory phenotype (SASP) of endothelium and SMCs. The secretion of various senescence factors activates the vasDCs, which produce signals recruiting T cells and monocytes to the vessel wall. Monocytes, activated by the inflammatory environment and T cell signaling, differentiate into macrophages. The further activation of macrophages, by the inflammatory environment/T cells, induces their fusion and the formation of multinuclear giant cells (GMCs). The GMCs over-produce proteolytic enzymes, which digest the extracellular matrix and disrupt the vessel wall integrity.

## 4. Giant Multinucleated Cells in Aging Gonads

In mammals, the first system showing signs of physiological aging is the female reproductive system. Although an age-related decline in fertility is mainly attributable to the declining quality of oocytes, the inflammaging of the ovarian tissue plays a vital role in this process. The oocyte environment consists of a stromal extracellular matrix (ECM), smooth muscle cells, endothelial cells, fibroblasts, and various immune cells. Studies of the ovaries from reproductively old mice showed excessive fibrosis of the ovarian stroma, which correlated with the overexpression of inflammatory cytokines and factors, as well as the presence of macrophage-derived multinucleated giant cells in the ovarian stroma [44,45]. It is known that macrophage fusion into giant cells is a hallmark of chronic inflammation. Thus, an inflammatory environment in the ovarian stroma may induce macrophage fusion. Macrophage fusion enhances phagocytosis and the degradation of large extracellular targets [34]. Although the exact function of giant cells in aged ovaries remains unknown, they may be involved in the degradation of large fibrotic regions or the removal of cellular debris accumulating in the ovary because of multiple ovulatory cycles and atresia of the follicles during the reproductive life span [44,45]. Recently, Foley et al. [45] suggested that macrophage-derived giant cells, absent in reproductively young mice, and their secretomes, are the markers and sources of inflammation in the aging ovary. They can also be a potential target in treatments promoting reproductive longevity.

## 5. Can Senescent Multinucleated Giant Cells Initiate Cancer?

**Figure 2:**
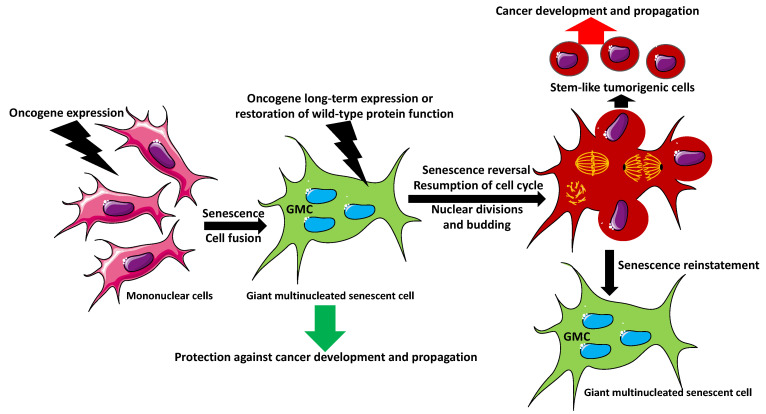
Reversal of senescence in giant multinucleated cells in cancer.

The oncogene-induced senescence (OIS) and accompanying bi- or multinucleated giant cell phenotype are well-known anti-cancer protection mechanisms and barriers to cancer progression. The senescence of premalignant cells blocks both tumorigenesis and tumor progression of the established tumors. By being arrested in the cell cycle, senescent giant multinuclear cells cannot propagate and generate tumors, despite having oncogenic mutation(s) [46,47,48,49]. This scenario is only valid if the senescence is irreversible, as we always assumed. However, studies show that, under certain conditions, OIS might be reversible. Examples of OIS inducers in various cell types are the proteins from the small GTPase Ras superfamily, which regulate cell divisions. Mutations in Ras proteins are among the most common genetic defects observed in human cancers. GTPase H-Ras (Harvey Rat sarcoma virus or transforming protein p21) regulates cell division in response to growth factors. H-Ras induces senescence of human fibroblasts and melanoma cells by inhibiting ribonucleotide reductase regulatory subunit M2 (RRM2), which regulates deoxyribonucleotide triphosphate (dNTP) metabolism. The low level of RRM2 induces cell-cycle arrest and senescence. However, the experimental addition of exogenous nucleosides decreases the formation of heterochromatin SAHF; suppresses the expression of senescence markers SA-β-gal, p16, and p21; and reverses senescence. Interestingly, the repeated depletion of nucleosides from these cells draws them into senescence again [46]. Another study showed that, not only the senescent multinucleated melanocytes can overcome senescence, but they become the source of highly aggressive mononucleated stem-like cells, which initiate tumor development and progression [47]. This study showed that N-Ras expression in mouse melanocytes induces senescence and multinucleated phenotypes. However, the long-term expression of N-Ras, using a doxycycline-inducible N-Ras vector, caused asynchronous cytokinesis in the multinucleated cells, resulting in the budding of small, mononuclear, proliferating cells called the anoikis (apoptosis induced by the loss of attachment to the substrate)-resistant (AR) cells. Interestingly, the parental cells remained multinuclear (Figure 2). These newly formed proliferating AR cells were highly tumorigenic both in vitro and in vivo. The gene expression analysis of AR cells showed that they expressed stem cell markers, such as the transcription factor Homeobox protein NANOG that supports stemness, self-renewal, and pluripotency by suppressing cell determination factors [47]. The formation of ARs is an example of neosis, an escape of cells from senescence, leading to neoplastic transformation and progression of cancer [48]. Interestingly, AR cells share stem-cell-like features with the progeny of polyploid giant cancer cells [49], indicating that multinucleated giant senescent cells are similar in their pro-tumorigenic potential to the polyploid cancer cells.

The expression of oncogenes, such as mutated Ras, induces cell-cycle arrest, senescence, and a fusion of mononuclear cells into multinucleated senescent cells. The senescence of premalignant cells serves as a protection mechanism against cancer progression. The senescent giant multinuclear cells (GMCs) cannot propagate and generate tumors, despite having oncogenic mutation(s). However, in certain circumstances, such as a long-term expression of the oncogene or an experimental restoration of the wild-type function of the mutated protein, GMCs overcome senescence and reactivate the cell cycle. The asynchronous nuclear divisions of GMCs, followed by the cytoplasm budding, produce stem-like, highly tumorigenic cells, which divide and metastasize. The parental GMC remains multinuclear and reverses to senescence.

## 6. Multinucleation in Cancer

Experimental and clinical data indicate that the cell fusion and formation of multinucleated cells in cancer are not only caused by changes in intrinsic factors, such as pathways and organelle dysfunction, cytoskeleton, and cytokinesis defects, but also by tumor microenvironment, and oncoviruses [50,51,52]. The fusion between cancer cells and mesenchymal stem cells, macrophages, endothelial cells, epithelial cells, and fibroblasts has been observed within various tumors [50,51]. The fusion between melanocytes and macrophages creates melanoma cells [53]. Multinucleated cancer cells can also derive from endomitosis, i.e., endoreplication coupled with karyokinesis without cytokinesis. Another recently described mechanism of multinucleation is the formation of “pregnant” P1 tumor cells, which intracytoplasmically generate and breed daughter cells [54]. Eventually, the daughter cells are expelled from a maternal cell by cytoplasm contraction. Maternal cells not only survive the birth process but can persist in producing many generations of daughter cells. Additionally, pregnant cells can transfer the progeny to the neighboring cells horizontally via tunneling tubes [54]. This process represents an entirely novel mechanism of tumor cell invasion and spreading.

## 7. Molecular Promoters and Repressors of Aging and Growth—A Lesson from a Worm and Fly

One of the principles of evolutionary biology is the tradeoff in the allocation of metabolic resources between the germline and soma. A high metabolic/nutritional investment in the reproduction and formation of the gametes depletes resources that are otherwise available for the somatic tissues and organs, thus affecting body size and longevity. Ample experimental and clinical data support this principle. Historically, the ancient Greek philosopher, Aristotle, noted the large size of mammals whose gonads were removed before puberty. Ancient Chinese, Ottoman Empire, and Italian writings described the large body size and very long limbs in castrated boys (eunuchs), the phenotype observed by modern medicine in hypogonadal patients [55]. Studies in the worm, *Caenorhabditis elegans*, showed that the laser-beam ablation of the precursor cells of the germline causes the gigantism [56]. Thus, the germline is the source of a signal suppressing growth. The germline also produces signals that repress longevity. The removal of the germline precursor cells extends the worm’s lifespan by 60% [57]. A germline-emitted signals affect the activity of an insulin/IGF-1 (insulin-like growth factor) pathway. Mutants with a reduced activity of the insulin/IGF-1-receptor homolog DAF-2 have a double lifespan, and their longevity requires the activity of transcription factor DAF-16, a member of the FOXO (forkhead box transcription factor O) family. The insulin/IGF pathway is also known to regulate longevity and aging in other species, including mammals [58,59,60,61,62]. Lin et al. [63] showed that the DAF-2 acts through a conserved phosphatidylinositol 3-kinase (PI 3-kinase)/Akt pathway. Mutations in this pathway inhibit worms’ aging and double the lifespan. DAF-2 signaling induces phosphorylation of DAF-16 by AKT activity, which, in turn, shortens the lifespan [63]. Studies combining the microarray expression screens of *C. elegans* strains with and without DAF-16 showed that DAF-16 activated 189 genes and repressed 122. The subsequent silencing of these genes using RNAi increased longevity by the upregulation of stress and antimicrobial response, metabolic stress, including ubiquitin-mediated protein turnover genes; the downregulation of the yolk protein vitellogenin gene, and a variety of other genes, which probably act cumulatively to shorten the lifespan [62,64]. Studies of Drosophila also showed that the overexpression of stress-response genes, such as hsp70, DNA repair gene mei-41, the loss of function of the ecdysone receptor Dts3, the insulin receptor (InR) Chico, and the type of the nutrition extend the lifespan [65,66,67]. Studies in humans and rodents showed that the dysregulation of nutrient metabolism caused by insulin resistance results in obesity and the chronic inflammation of adipose tissue. These studies also showed that insulin levels affect the expression of macrophage-specific genes and increase macrophage infiltration into the adipose tissue. Eventually, chronic inflammation related to macrophage activity induces multinucleated giant cells, which may contribute to adipocyte lipolysis and the pathogenesis of insulin resistance, obesity, and aging [45,68].

Studies in mammals showed that the glucose level (hyperglycemia and hyperinsulinemia) and the insulin/insulin-like growth factor 1 (IGF-1) pathway-related molecules, play an essential role in aging. The lower levels of IGF-1 resulting from the calory restricted diet, and antidiabetic drugs increase longevity [69].

Recent studies in flies indicate that one of the promoters of giant cell formation is autophagy. Autophagy is a degradation process conserved in all species. It encloses molecules and organelles inside double-membrane vesicles called autophagosomes, delivering them to lysosomes for degradation [70]. Autophagy is a common process in development, differentiation, and tissue remodeling and is activated during wound healing in Drosophila and other species [71]. Studies in Drosophila showed that autophagy can also have cytoprotective functions [72] and induces formation of multinucleated giant cells [73]. Drosophila larvae have elevated levels of autophagy in the epidermis, both during development and epidermal wound healing [73]. Autophagy induces cell membrane breakdown, facilitating cell fusion and the formation of giant syncytial cells [73]. Other studies showed that autophagy is necessary to form syncytiotrophoblast in the mammalian placenta and myoblast fusion [74,75]. However, further studies are required to establish how autophagy promotes cell fusion.

Thus, the aging-controlling molecules and mechanisms conserved in different model systems are probably also involved in human longevity. Studies in the model systems showed that longevity can be increased by targeting various senescence-inducing pathways [76]. In Drosophila, the increase of resistance to oxidative stress by the overexpression of free radical scavenger superoxide dismutase delayed aging and increased the life span of transgenic flies [77,78]. A similar correlation has also been described in C. elegans worm, where long-lived mutant worms had increased superoxide dismutase and catalase activity [79]. Studies also showed that drugs mimicking catalase and superoxide dismutase activities extended worms’ life span [80].

Thus, the components of various senescence-inducing pathways could be a potential target of anti-aging interventions in humans. Additionally, further studies of the role of multinucleated giant cells and mechanisms regulating their senescence should contribute to our understanding of aging and cancer development.

## 8. Conclusions

Senescent multinucleated giant cells and their secretomes are the markers and source of inflammation in aging arteries and gonads. Multinucleated giant cells are involved in the development of age-related diseases and the progression of cancer. Targeting the multinucleated cells and senescence-inducing factors and pathways can be used to increase longevity for the prevention and treatment of age-related diseases and cancer.

## Data Availability

Not applicable.

## Abbreviations

AKT	serine/threonine-specific protein kinases family
Bcl-2	B-cell lymphoma-2 protein
cGAS	DNA-sensing receptor cyclic GMP–AMP synthase
DAF-2	the insulin-like growth factor 1 receptor
DAF-16/FOXO	forkhead box transcription factor O
DC	dendritic cell
DDR	DNA-damage response pathway
dNTP	deoxyribonucleotide triphosphate
E2F	E2 family transcription factor
ECM	extracellular matrix
FOXO	forkhead box transcription factor O
GCA	giant cell arteritis
GMC	giant multinucleated cell
GM-CSF	granulocyte-macrophage colony stimulating factor
GRO-a	growth-regulated protein alpha
Hsp70	heat shock protein 70
ICAM-1	intercellular adhesion molecule 1
IFN γ	interferon γ
IL-1a	interleukin 1 alpha (IL-1α) also known as hematopoietin 1
IL-6	interleukin 6
IL-8	interleukin 8, a chemoattractant cytokine
IL-15	interleukin 15
IGF	insulin-like growth factor
MIP-1a	macrophage inflammatory protein-1 alpha (MIP-1 alpha)/CCL3
OXPHOS	oxidative phosphorylation pathway
pRB	retinoblastoma protein
PI 3-kinase	phosphatidylinositol 3-kinase
p16Ink4a/RB	cyclin-dependent kinase inhibitor/retinoblastoma tumor suppressor pathway
p53/p21CIP1	tumor protein p53/cyclin-dependent kinase inhibitor 1
RAS	rat sarcoma virus small GTPase
HRAS	Harvey rat sarcoma virus or transforming protein p21, small GTPase
ROS	reactive oxygen species
RRM2	ribonucleotide reductase regulatory subunit M2
SA-βgal	senescence-associated beta-galactosidase
SAHFs	senescence-associated heterochromatin foci
SASP	senescence-associated secretory phenotype
STING	stimulator of interferon genes
vasDC	vascular dendritic cells
VEGF	vascular endothelial growth factor
